# A genome-wide enhancer/suppressor screen for *Dube3a* interacting genes in *Drosophila melanogaster*

**DOI:** 10.1038/s41598-019-38663-y

**Published:** 2019-02-20

**Authors:** Kevin A. Hope, Addison McGinn, Lawrence T. Reiter

**Affiliations:** 1 Department of Neurology, University of Tennessee Health Science Center, Memphis, TN USA; 2 Integrated Biomedical Science Program, University of Tennessee Health Science Center, Memphis, TN USA; 3 The University of Oklahoma College of Medicine, Oklahoma City, OK USA; 4 Department of Pediatrics, University of Tennessee Health Science Center, Memphis, TN USA; 5 Department of Anatomy and Neurobiology, University of Tennessee Health Science Center, Memphis, TN USA

## Abstract

The genetics underlying autism spectrum disorder (ASD) are complex. Approximately 3–5% of ASD cases arise from maternally inherited duplications of 15q11.2-q13.1, termed Duplication 15q syndrome (Dup15q). 15q11.2-q13.1 includes the gene *UBE3A* which is believed to underlie ASD observed in Dup15q syndrome. UBE3A is an E3 ubiquitin ligase that targets proteins for degradation and trafficking, so finding UBE3A substrates and interacting partners is critical to understanding Dup15q ASD. In this study, we take an unbiased genetics approach to identify genes that genetically interact with *Dube3a*, the *Drosophila melanogaster* homolog of *UBE3A*. We conducted an enhancer/suppressor screen using a rough eye phenotype produced by *Dube3a* overexpression with *GMR-*GAL4. Using the DrosDel deficiency kit, we identified 3 out of 346 deficiency lines that enhanced rough eyes when crossed to two separate *Dube3a* overexpression lines, and subsequently identified *IA2*, *GABA-B-R3*, and *lola* as single genes responsible for rough eye enhancement. Using the FlyLight GAL4 lines to express *uas-Dube3a* + *uas-GFP* in the endogenous *lola* pattern, we observed an increase in the GFP signal compared to *uas-GFP* alone, suggesting a transcriptional co-activation effect of *Dube3a* on the *lola* promoter region. These findings extend the role of *Dube3a/UBE3A* as a transcriptional co-activator, and reveal new *Dube3a* interacting genes.

## Introduction

Autism spectrum disorder (ASD) has a prevalence of 1 in 68^1^, and is characterized by social communication deficits and repetitive behaviors^2^. While the underlying genetic causes of autism are complex, recurrent copy number variants associated with ASD can provide insight into the genetic mechanism as they are consistently observed at high rates among ASD individuals^3–6^. One such disorder results from maternally derived duplications of chromosome 15q11.2-q13.1, termed Duplication 15q (Dup15q) syndrome and the majority of Dup15q individuals meet the criteria for ASD^7^. A single gene located within 15q11.2-q13.1, the E3 ubiquitin ligase *UBE3A*, has been implicated in driving ASD features of Dup15q^8,9^. Identification of UBE3A substrates and interacting proteins, however, has been a difficult task^10^.

Using the GAL4/UAS system^11^, our lab has constructed a Dup15q syndrome model in *Drosophila melanogaster* through overexpression of *Dube3a* (the fly *UBE3A* homolog) to investigate molecular consequences of elevated levels of *Dube3a* in the fly nervous system. This study supplements our previous proteomics screen^12^ by taking an unbiased genetics approach to identify genes that interact with *Dube3a*. Here, we performed an enhancer/suppressor screen, using a rough eye phenotype produced by over-expression of *Dube3a* with *GMR-*GAL4, to identify new *Dube3a* interacting genes. Using the DrosDel deficiency kit that covers 65.2% of the fly genome^13^, and subsequent *uas-RNAi* lines, we found three new *Dube3a* interacting genes: *GABA-B-R3, IA2*, and *lola*.

## Results

### GABA-B-R3, IA2, and lola enhance the GMR > Dube3a rough eye phenotype

**Figure 1:**
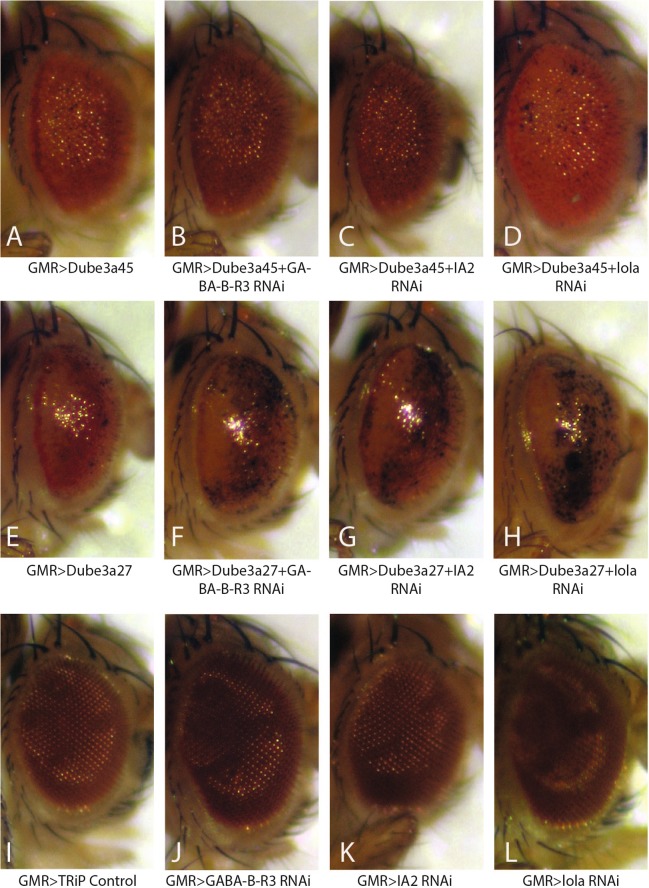
Identification of enhancers of the rough eye phenotype generated by *Dube3a* overexpression with *GMR*-GAL4. The first column represents control fly eyes, indicating the mild rough eye phenotype generated with *GMR* > *Dube3a45* (**A**) and the severe rough eye phenotype with *GMR* > *Dube3a27* (**E**). Columns 2–4 are representative images of eyes of the enhancement observed with *GABA-B-R3-RNAi* (**B,F**), *IA2-RNAi* (**C**,**G**), and *lola-RNAi* (**D**,**H**), respectively. Note the increase in necrosis observed in the RNAi lines crossed to *GMR* > *Dube3a27* (**E**–**H**). No rough eye phenotypes were observed with the *GMR* > *TRiP-Control* line or any *uas-RNAi* line alone (**I**–**L**).

We utilized two *GMR* > *Dube3a* lines in this screen that were previously characterized to identify modifiers of the rough eye phenotype^14^. The line *GMR* > *Dube3a45* has moderate *Dube3a* overexpression that causes a mild rough eye phenotype, while *GMR* > *Dube3a27* has higher *Dube3a* expression levels and causes a more severe eye phenotype with underlying necrosis. Out of the 346 DrosDel lines, we found that Df(2 L)ED62, Df(2 L)ED105, and Df(2 R)ED2076 enhanced rough eyes when crossed to both *GMR* > *Dube3a45 and GMR* > *Dube3a27*. Next we used a combination of smaller deficiency lines eliminating smaller segments of the genome within each DrosDel line and *uas-RNAi* lines to identify the specific genes within each region that, when knocked down, enhanced rough eyes produced by *Dube3a* overexpression (Table 1). We found that expression of RNAi for *GABA-B-R3, IA2*, and *lola* enhanced rough eyes in both *GMR* > *Dube3a45* and *GMR* > *Dube3a27*, yet RNAi expression for these three genes alone had no effect on eye morphology (Fig. 1). These experiments suggest that *Dube3a* interacts genetically with *GABA-B-R3*, *IA2*, and *lola* as knockdown of these genes enhances the rough eye produced by *Dube3a* overexpression.

**Table 1:** Fly lines used in the enhancer/suppressor screen to identify single genes acting as enhancers/suppressors of the rough eye phenotype.

Primary Screen	Secondary Screen	Tertiary Screen	Gene Identified
*Df(2* *L)ED62* (+)	*Df(2* *L)BSC107* (−)	*Ds-RNAi* (−)	*GABA-B-R3*
	*Df(2* *L)EXEL8003* (−)	*EAAT2-RNAi* (−)	
	*Df(2* *L)EXEL6002* (+)	*GABA-B-R3-RNAi* (+)	
*Df(2* *L)ED105* (+)	*Df(2* *L)EXEL6004* (−)	*CG4341-RNAi* (−)	*IA2*
	*Df(2* *L)EXEL6003* (+)	*drongo-RNAi* (−)	
	*Df(2* *L)ED108* (−)	*IA2-RNAi* (+)	
*Df(2* *L)ED2076* (+)	*Lola-RNAi* (+)		*Lola*
	*PSQ-RNAi* (−)		
	*MAT1-RNAi* (−)		
	*STAN-RNAi* (−)		

Primary screen indicates the initial DrosDel deficiency line identified as an enhancer. Secondary screen indicates lines used to narrow the genes located within the identified region, followed by tertiary screen lines if necessary. The genes identified as enhancers are listed in the right most column. (+) indicates rough eye enhancement, (−) indicates no effect.

### Overexpression of *Dube3a* in the Endogenous *lola* Expression Pattern Enhances GFP Reporter Signal

**Figure 2:**
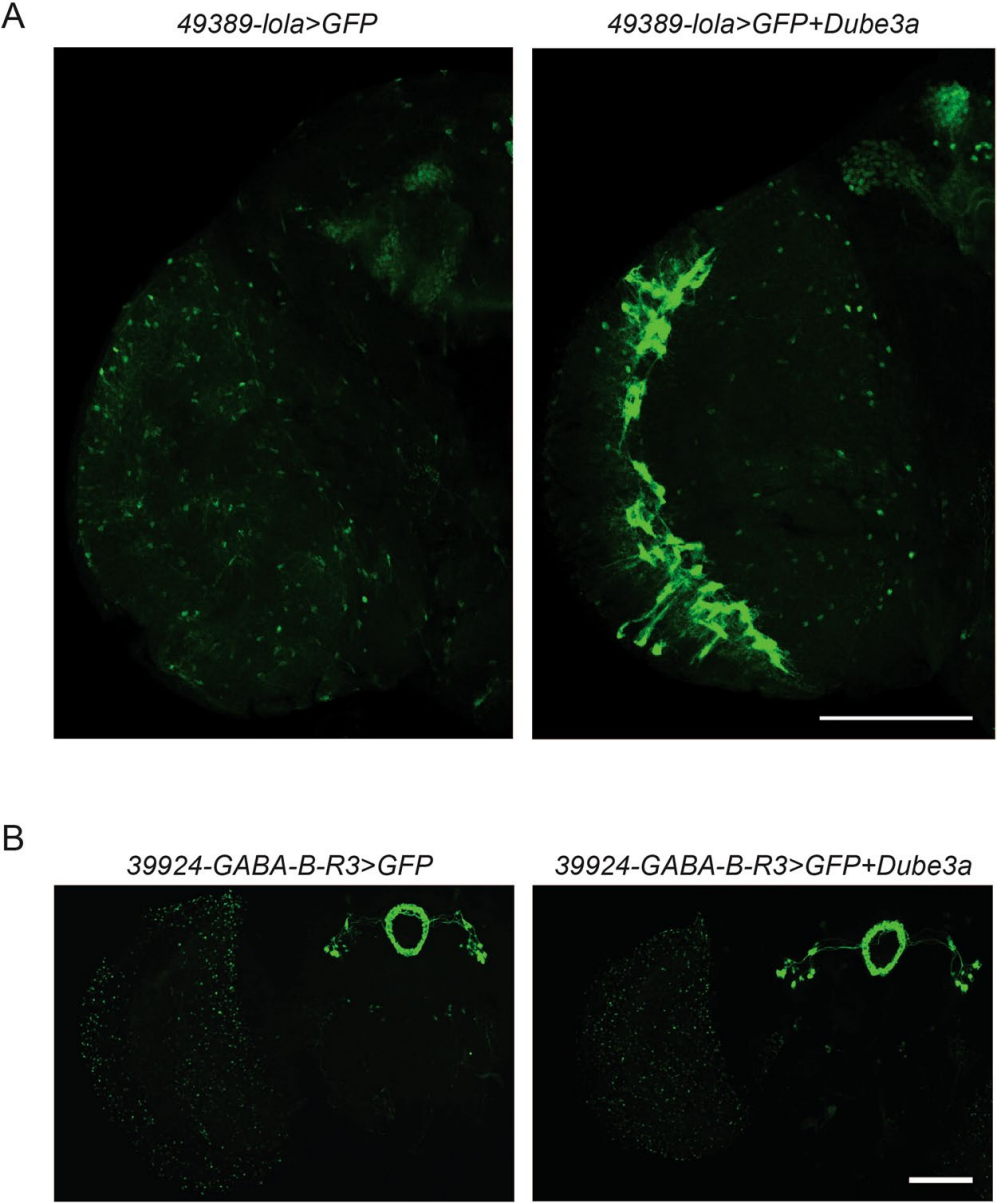
Increased expression of the GFP reporter in *49389-lola* > *GFP* + *Dube3a* flies. (**A**) Expression of the GFP reporter with *49389-lola* > *GFP* revealed a relatively diffuse expression pattern throughout the fly optic lobe with some expression in the mushroom body (left). In *49389-lola* > *GFP* + *Dube3a* flies we observed a dramatic increase in GFP levels, particularly cells in the optic lobe (right). (**B**) Expression of the *GFP* reporter with or without *Dube3a* in the pattern of *GABA-B-R3* with *39924-GABA-B-R3-GAL4* revealed no observable differences between *GFP* alone (left) and *GFP* + *Dube3a* (right). Scale bar is 100 µm.

Next, we used the FlyLight GAL4 collection^15^ to express *Dube3a* in the endogenous pattern of genes identified as *Dube3a* interactors with a GFP reporter. Each of these fly lines was generated by taking a small segment of DNA that lies upstream of the gene of interest and using it to control the expression of the GAL4 protein. When we expressed *GFP* + *Dube3a* in the pattern of *lola* with the line *49389-lola-GAL4* we observed a marked increase in the GFP signal in the optic lobe compared to GFP alone (Fig. 2A). However, when we expressed *GFP* + *Dube3a* in the patterns of *GABA-B-R3* we observed no appreciable difference between *GFP* alone and *Dube3a* + *GFP* signals (Fig. 2B), and no FlyLight GAL4 lines were available for *IA2*. These data suggest that Dube3a interacts with the DNA sequences upstream of *lola*, potentially through a transcriptional co-activation mechanism.

## Discussion

The primary function of the UBE3A E3 ligase is to identify and ubiquitinate substrate proteins^16,17^. The identification of *Dube3a/UBE3A* interacting partners, however, has been proven to be a difficult task. One hurdle to identifying UBE3A substrates may be due the transient nature of the interaction between UBE3A and its target protein. In this study we took an unbiased genetics approach using the tractable model organism *Drosophila melanogaster* and identified *IA2*, *GABA-B-R3*, and *lola* as genetic interactors with *Dube3a*.

The gene *IA2* encodes a receptor protein tyrosine phosphatase that facilitates secretion of insulin-like peptides in Drosophila^18^, and insulin signaling is important for proper eye development^19^. It is possible that in a *Dube3a* overexpression background, perturbation of insulin signaling through *IA2* knockdown further impairs eye development, suggesting that Dube3a interacts with proteins involved in insulin signaling pathways. *GABA-B-R3* encodes for a metabotropic GABA receptor that displays a unique expression profile compared to other Drosophila metabotropic GABA receptors^20^. GABA acts as an inhibitory neurotransmitter at GABA-B-R3 receptors through Go G-protein coupling and regulates circadian rhythm behavior^21^. Aside from the role of GABA-B-R3 in circadian rhythms, the function of *GABA-B-R3* is relatively unknown. How *Dube3a* and *GABA-B-R3* interact in the eye is unclear, however our data suggests that Dube3a interacts with the GABAergic system or G-protein coupled receptor function. *Lola* is a transcriptional repressor and subsets of *lola*-dependent genes are involved in eye development, cell death, and actin cytoskeleton regulation^22^. We previously demonstrated that Dube3a regulates the actin cytoskeleton^12^, implying that actin cytoskeleton disruption may play a role in the rough eye enhancement phenotype. While *IA2* has the strong human ortholog *PTPRN* with an 8/12 DIOPT score^23^, *GABA-B-R3* and *lola* do not have clear human orthologs. Nonetheless, insights into the basic biology of and the nature of interactions between the identified genes and *Dube3a* can still provide insight into the function of *Dube3a*/*UBE3A*.

We observed an increase in GFP signal in *49389-lola* > *GFP* + *Dube3a* flies compared to *49389-lola* > *GFP* alone flies. It is unlikely that Dube3a is acting on the UAS promoter region of GFP because the same GFP construct was used in both the *lola-GAL4* and *GABA-B-R3-GAL4* experiments. We only observed increased GFP expression with *lola-GAL4* (Fig. 2). Therefore, we hypothesize that the increased GFP signal is due to the transcriptional co-activation function of Dube3a, a phenomenon previously reported by our lab and others^24–26^, but largely unexplored. Our working model is that in *49389-lola* > *GFP* flies, the GAL4 protein binds to the *uas-GFP* sequence, driving expression of the GFP reporter. However, in *49389-lola* > *GFP* + *Dube3a* flies, the GAL4 protein binds to both the *uas-GFP* sequence and the *uas-Dube3a* sequence, causing both *GFP* and *Dube3a* to be expressed. If Dube3a is acting as a transcriptional co-activator, Dube3a could be looping back to the *49389-lola-GAL4* sequence and driving expression of more GAL4 protein. The increase in GAL4 protein is then further driving expression of *GFP*, resulting in a feedback loop causing the increased GFP fluorescence observed in *49389-lola* > *GFP* + *Dube3a* flies. An alternative mechanism could be the Dube3a-dependent degradation of a region specific *lola* repressor that leads to increased *GFP* expression, and this repressor is not present at the *GABA-B-R3* locus. While these hypotheses remain to be tested in further detail, the data presented here serves as a solid starting point since the DNA sequence used to drive GAL4 expression in the *49389-lola-*GAL4 line is known.

In conclusion, our lab utilized an unbiased enhancer/suppressor screen and identified *GABA-B-R3, IA2*, and *lola* as *Dube3a* interacting genes. Dube3a appears to have transcriptional co-activation activity within a subset of *lola* expressing cells which remains to be investigated further.

## Materials and Methods

### Fly Stocks

Flies were reared at 25 °C on standard Drosophila corn meal media. *GMR-*GAL4, *49389-lola*-GAL4, the DrosDel Deficiency Kit, *uas-lola-RNAi*, *uas-IA2-RNAi*, and *uas-GABABR3-RNAi*, along with other *uas-RNAi* lines were obtained from the Bloomington Drosophila Stock Center (for complete list of stocks, see Supplemental Table 1). The *uas-Dube3a* lines used in these experiments were described previously^14^. The line *uas-Dube3a45* has moderate *Dube3a* expression and produces a mild rough eye phenotype when crossed to *GMR-*GAL4, while *uas-Dube3a27* line has higher *Dube3a* overexpression with *GMR-*GAL4 and produces a severe rough eye phenotype with underlying necrosis^14^. Stock lines were made consisting of *GMR-*GAL4 + *uas-Dube3a45* or *GMR-*GAL4 + *uas-Dube3a27*, which were subsequently crossed to DrosDel deficiency lines. The *uas-GFP* lines was a gift from Dr. Cynthia Hughes.

### Enhancer Suppressor Screen

Male flies from the DrosDel deficiency kit were crossed to virgin female *GMR*-GAL4 + *uas-Dube3a45* and GAL4 + *uas-Dube3a27* flies at 25 °C. 3–5 days after eclosion, eyes were compared to *GMR* > *Dube3a45* and *GMR* > *Dube3a27* alone. Enhancement was defined as an increase in the following parameters: eye roughness, fusion of ommatidia, and/or underlying necrosis (yellow and black color). Any DrosDel line identified as an enhancer of the rough eye phenotype caused by Dube3a overexpression was independently scored by a second observer for confirmation. The flybase genome browser was used to identify secondary deficiencies and genes within each region that were subsequently tested for enhancement of rough eyes with *uas-RNAi* lines for individual genes located within the region. Images were captured on a PowerShot S50 (Canon) camera mounted to a Leica L2 compound dissecting microscope and processed with PhotoShop (Adobe). Adjustments to brightness and contrast were consistent across all images.

### Fly Brain Dissection and Imaging

3–5 day old flies were briefly anesthetized with CO_2_, heads were removed, and brains were dissected in phosphate buffered saline (PBS). Brains were fixed in 4% formaldehyde, washed with PBS, and mounted on glass slides with Vectashield Mounting media (Vector Labs H-1200). Images were acquired on a Zeiss 710 confocal microscope (Zeiss) located in the UTHSC Neuroscience Institute Imaging Core at 1024 × 1024 resolution with the detector gain and offset optimized to use the full 12-bit linear range. Microscope settings remained constant between control and experimental groups. GFP was excited using a 488 nm laser and emission was collected at 493–569 nm. Z-sections were acquired at 1 µm optical section thickness through the entirety of the brain. Images were acquired and z-stacks were transformed to maximum intensity projections using ZEN software (Zeiss).

## Supplementary information

Supplemental Table 1

## Data Availability

All data generated during this study are included in this manuscript, the supplemental materials, or can be made available from the corresponding author upon reasonable request.

## References

[1] Christensen DL (2016). Prevalence and Characteristics of Autism Spectrum Disorder Among Children Aged 8 Years–Autism and Developmental Disabilities Monitoring Network, 11 Sites, United States, 2012. MMWR Surveill Summ.

[2] American Psychiatric Association. *Diagnostic and Statistical Manual of Mental Disorders: DSM-5.5th edn*. 5th edn (2013).

[3] Sebat J (2007). Strong association of de novo copy number mutations with autism. Science.

[4] Pinto D (2010). Functional impact of global rare copy number variation in autism spectrum disorders. Nature.

[5] Moreno-De-Luca D (2013). Using large clinical data sets to infer pathogenicity for rare copy number variants in autism cohorts. Mol Psychiatry.

[6] Noh HJ (2013). Network topologies and convergent aetiologies arising from deletions and duplications observed in individuals with autism. PLoS Genet.

[7] Finucane, B. M. *et al*. In *GeneReviews(R)* (eds R. A. Pagon *et al*.) (2016).

[8] Hogart A, Wu D, LaSalle JM, Schanen NC (2010). The comorbidity of autism with the genomic disorders of chromosome 15q11.2-q13. Neurobiol Dis.

[9] Urraca N (2013). The interstitial duplication 15q11.2-q13 syndrome includes autism, mild facial anomalies and a characteristic EEG signature. Autism Res.

[10] LaSalle JM, Reiter LT, Chamberlain SJ (2015). Epigenetic regulation of UBE3A and roles in human neurodevelopmental disorders. Epigenomics.

[11] Duffy JB (2002). GAL4 system in Drosophila: a fly geneticist’s Swiss army knife. Genesis.

[12] Jensen L, Farook MF, Reiter LT (2013). Proteomic profiling in Drosophila reveals potential Dube3a regulation of the actin cytoskeleton and neuronal homeostasis. PLoS One.

[13] Cook RK (2012). The generation of chromosomal deletions to provide extensive coverage and subdivision of the Drosophila melanogaster genome. Genome Biol.

[14] Reiter LT, Seagroves TN, Bowers M, Bier E (2006). Expression of the Rho-GEF Pbl/ECT2 is regulated by the UBE3A E3 ubiquitin ligase. Hum Mol Genet.

[15] Pfeiffer BD (2008). Tools for neuroanatomy and neurogenetics in Drosophila. Proc Natl Acad Sci USA.

[16] Kim HC, Huibregtse JM (2009). Polyubiquitination by HECT E3s and the determinants of chain type specificity. Mol Cell Biol.

[17] Zheng, N. & Shabek, N. Ubiquitin Ligases: Structure, Function, and Regulation. *Annu Rev Biochem*, 10.1146/annurev-biochem-060815-014922 (2017). 10.1146/annurev-biochem-060815-014922 28375744

[18] Kim J (2008). Drosophila ia2 modulates secretion of insulin-like peptide. Comp Biochem Physiol A Mol Integr Physiol.

[19] Huang H (1999). PTEN affects cell size, cell proliferation and apoptosis during Drosophila eye development. Development.

[20] Mezler M, Muller T, Raming K (2001). Cloning and functional expression of GABA(B) receptors from Drosophila. Eur J Neurosci.

[21] Dahdal D, Reeves DC, Ruben M, Akabas MH, Blau J (2010). Drosophila pacemaker neurons require g protein signaling and GABAergic inputs to generate twenty-four hour behavioral rhythms. Neuron.

[22] Gates MA, Kannan R, Giniger E (2011). A genome-wide analysis reveals that the Drosophila transcription factor Lola promotes axon growth in part by suppressing expression of the actin nucleation factor Spire. Neural Dev.

[23] Wang J (2017). MARRVEL: Integration of Human and Model Organism Genetic Resources to Facilitate Functional Annotation of the Human Genome. Am J Hum Genet.

[24] Nawaz Z (1999). The Angelman syndrome-associated protein, E6-AP, is a coactivator for the nuclear hormone receptor superfamily. Mol Cell Biol.

[25] Ferdousy F (2011). Drosophila Ube3a regulates monoamine synthesis by increasing GTP cyclohydrolase I activity via a non-ubiquitin ligase mechanism. Neurobiol Dis.

[26] El Hokayem J, Nawaz Z (2014). E6AP in the brain: one protein, dual function, multiple diseases. Mol Neurobiol.

